# High Dose Versus Low Dose Syngeneic Hepatocyte Transplantation in *Pex1*-G844D NMRI Mouse Model is Safe but Does Not Achieve Long Term Engraftment

**DOI:** 10.3390/cells10010040

**Published:** 2020-12-30

**Authors:** Tanguy Demaret, Jonathan Evraerts, Joachim Ravau, Martin Roumain, Giulio G. Muccioli, Mustapha Najimi, Etienne M. Sokal

**Affiliations:** 1 Laboratoire d’Hépatologie Pédiatrique et Thérapie Cellulaire, Unité PEDI, Institut de Recherche Expérimentale et Clinique (IREC), Université Catholique de Louvain (UCLouvain), 1200 Brussels, Belgium; jonathan.evraerts@uclouvain.be (J.E.); joachim.ravau@uclouvain.be (J.R.); mustapha.najimi@uclouvain.be (M.N.); etienne.sokal@uclouvain.be (E.M.S.); 2 Bioanalysis and Pharmacology of Bioactive Lipids Research Group (BPBL), Louvain Drug Research Institute (LDRI), Université Catholique de Louvain (UCLouvain), 1200 Brussels, Belgium; martin.roumain@uclouvain.be (M.R.); giulio.muccioli@uclouvain.be (G.G.M.)

**Keywords:** peroxisome biogenesis disorder, Zellweger spectrum disorder, oxysterols, mouse model, PEX1 p.Gly843Asp, PEX1 c.2528G>A

## Abstract

Genetic alterations in *PEX* genes lead to peroxisome biogenesis disorder. In humans, they are associated with Zellweger spectrum disorders (ZSD). No validated treatment has been shown to modify the dismal natural history of ZSD. Liver transplantation (LT) improved clinical and biochemical outcomes in mild ZSD patients. Hepatocyte transplantation (HT), developed to overcome LT limitations, was performed in a mild ZSD 4-year-old child with encouraging short-term results. Here, we evaluated low dose (12.5 million hepatocytes/kg) and high dose (50 million hepatocytes/kg) syngeneic male HT via intrasplenic infusion in the *Pex1*-G844D NMRI mouse model which recapitulates a mild ZSD phenotype. HT was feasible and safe in growth retarded ZSD mice. Clinical (weight and food intake) and biochemical parameters (very long-chain fatty acids, abnormal bile acids, etc.) were in accordance with ZSD phenotype but they were not robustly modified by HT. As expected, one third of the infused cells were detected in the liver 24 h post-HT. No liver nor spleen microchimerism was detected after 7, 14 and 30 days. Future optimizations are required to improve hepatocyte engraftment in *Pex1*-G844D NMRI mouse liver. The mouse model exhibited the robustness required for ZSD liver-targeted therapies evaluation.

## 1. Introduction

Zellweger spectrum disorders (ZSD) are caused by genetic alterations in *PEX* genes encoding peroxins [1]. Peroxin deficiency lead to peroxisome biogenesis disorder and metabolic imbalance [2]. Metabolites catabolized into peroxisomes (i.e., branched-chain fatty acids (BCFA: phytanic and pristanic acids), very long-chain fatty acids (VLCFA: C_22_, C_24_, C_26_), C_27_ bile acids (BA) precursors, etc.) accumulate into plasma and tissues, and compounds synthetized in the peroxisomes are deficient (i.e., plasmalogens, C_24_ BA, etc.) [3]. Most mild ZSD patients survive into childhood, however no validated treatment is able to impact their progressive hepatic dysfunction and developmental delay [4]. In this context, we and others have obtained proof-of principle that liver-targeted therapies such as living donor liver transplantation (LT) and hepatocyte transplantation (HT) can modify disease progression in mild ZSD patients [5,6,7,8].

Because organ shortage is a major hurdle to pediatric LT, several techniques have been developed to increase organ availability (living donor LT, split-liver, etc.) [9,10,11]. Yet, parenchymal function is often preserved in liver-based metabolic diseases making it difficult for these patients to progress on LT waiting list [12]. Hepatocyte transplantation (HT) was developed to overcome LT limitations (i.e., heaviness, cost, organ availability, etc.). HT consists in isolating hepatocytes from liver not suitable for LT and infusing them to a recipient with or without cryopreservation [13]. First pediatric HT procedure performed in Europe was for a four-year-old child affected by mild ZSD [8]. It led to encouraging biochemical results.

With an estimated incidence rate close to 1/100,000, ZSD meet rare disease criteria [14,15,16]. Since robust placebo-controlled clinical trials are out of the scope in these diseases, treatments are mainly evaluated in animal models which allow rigorous controlled conditions [17]. The *Pex1*-G844D NMRI mouse model we recently developed displays biochemical and hepatic alterations affecting mild ZSD patients [18]. Interestingly, its easy breeding and robustness make it suitable for preclinical studies and intervention like HT.

In order to strengthen liver-directed therapies in mild ZSD therapeutic approaches, we evaluated two HT protocols in *Pex1*-G844D NMRI mouse model. We followed clinical and biochemical parameters along with liver hepatocyte engraftment to monitor HT impacts on mild ZSD phenotype.

## 2. Materials and Methods

### 2.1. Mice Care, Breeding and Genotyping

Heterozygous *Pex1*-G844D NMRI mice from in-house colony were bred in a specific-pathogen-free environment, and in individually ventilated cages with 12 h light/12 h dark cycle [18]. Wild-type and heterozygous mice exhibited no phenotype difference and were used as control mice. Mice had unrestricted access to food (Carfil, Oud-Turnhout, Belgium, Mice Breeding diet) and water, and were genotyped as described earlier [18]. All animal experiments were carried out in accordance with the EU Directive 2010/63/EU for animal experiments and approved by the Ethical Committee for Animal Experimentation at the Health Science Sector, UCLouvain, Brussels, Belgium (2017/UCL/MD/006).

### 2.2. Hepatocyte Transplantation

**Figure 1:**
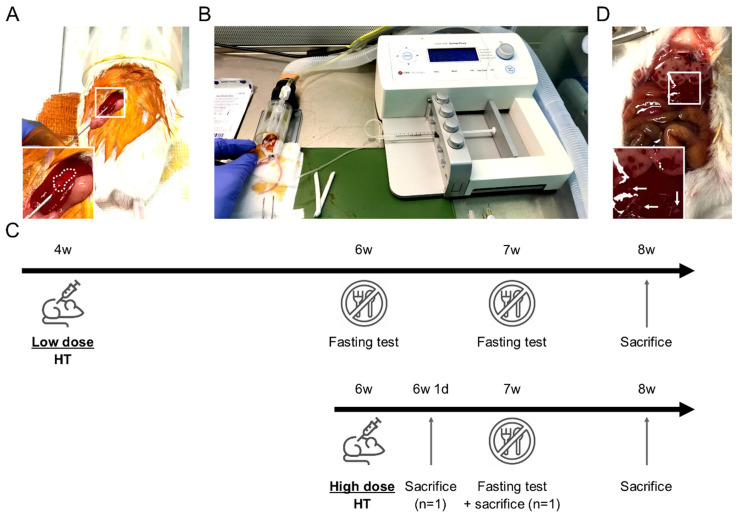
Mouse hepatocyte transplantation (HT) setup and HT protocols description. (**A**) Mouse in right lateral decubitus with intra-splenic 25 G needle insertion. Note the discolored spleen area inside the dotted line confirming the correct intra-splenic infusion flushing erythrocytes. (**B**) Device setup used for HT: syringe press on the right, heating pad (green) and anesthesia tunnel delivering isoflurane mixed with oxygen and collecting exhaled gases. (**C**) Schematic description of the two HT protocols including fasting tests, sacrifices for microchimerism evaluation and samples collection at the end of the study. (**D**) Macroscopic liver evaluation 24 h after high dose HT highlighting large necrosis areas in right and left median lobes. Left lateral lobe displayed smaller necrosis areas (white arrows). Inserts show magnification of the boxed areas.

Male mouse hepatocytes were isolated from control littermate by collagenase P (Roche, Indianapolis, IN, USA, 11213873001) as we previously described [19]. Cells were suspended at 1.25 or 5 million hepatocytes/mL in *N*-acetylcysteine 4% (NAC, Lysomucyl, Zambon, Bruxelles, Belgium) in phosphate buffered saline (PBS, Lonza, Walkersville, MD, USA, 15-512F). NAC was shown to inhibit de procoagulant activity of isolated hepatocytes [20]. Two HT protocols were evaluated based on 12.5 or 50 million cells/kg (10 µL/g of body weight), further referred as low dose HT and high dose HT, respectively. They were defined to deliver a number of hepatocytes representing ~0.1 and ~0.5% of the recipient liver, respectively. In the low dose protocol, we infused a relatively limited number of hepatocytes to minimally trigger the mechanisms responsible for their clearance (*vide* Discussion). Younger mice (4 weeks) were transplanted in order to take benefit from the proliferation signals present in the growing liver. The longer follow-up (4 weeks) post-HT was designed to allow the proliferation of the infused hepatocytes. In the high dose protocol, older larger mice (6 weeks) received a higher (absolute and relative) number of hepatocytes to evaluate the effect of engrafted and circulating hepatocytes. This was combined with a shorter follow-up (2 weeks) to prevent the consequences of hepatocyte loss. HT was performed in the morning based on a modified protocol from [21]. Briefly, under isoflurane anesthesia, freshly isolated hepatocytes were infused by intrasplenic injection through 25 G needle at 50 µL/minute with a syringe press (CMA 400 microdialysis, Kista, Sweden) (Figure 1A,B). Control mice received 100 µL PBS-NAC. All mice received subcutaneous 20 mL/kg warmed NaCl 0.9% (Mini-Plasco NaCl 0.9%, B. Braun, Diegem, Belgium) along with 0.1 mg/kg buprenorphine (Temgesic, Schering-Plough, Kenilworth, NJ, USA) once before the procedure and then twice a day for 2 days. Mice groups and follow-up are presented (Table 1, Figure 1C). Two ZSD mice infused with high dose HT were sacrificed earlier to evaluate microchimerism longitudinally (Figure 1C). These mice were excluded from HT outcomes analysis.

### 2.3. Clinical Evaluation and Glycemia

Food and mouse weight were measured once a week. Morning glycemia was measured on tail vein blood droplet using glucometer (FreeStyle Precision Neo, Abbott, Princeton, NJ, USA). Mice were fasted 6 h in a clean cage with access to water before glycemia quantification to evaluate their glycemic response to fast (Figure 1C).

### 2.4. Blood and Liver Collection

Four or 2 weeks post-HT (Table 1), mice were anesthetized by intraperitoneal injection with a solution containing ketamine and xylazin at final concentration of 100 mg/kg and 10 mg/kg, respectively. Plasma and liver lobes were collected as described previously [18]. Briefly, plasma was aliquoted and snap frozen in liquid nitrogen. Left lateral lobe was fixed in formaldehyde 4% (VWR, Lille, France, 11699408) overnight and embedded in paraffin for histological analysis. Left medial lobe (LML) was snap frozen in liquid nitrogen and stored at −80 °C for biochemical and microchimerism analysis.

### 2.5. Peroxisomal Markers Quantification

BCFA, VLCFA, oxysterols, BA and pipecolic acid were quantified as described earlier [18].

### 2.6. Histological Analyses

Formalin-fixed paraffin-embedded left lateral liver lobes were cut into 5 µm thick wide sections and processed for hematoxylin and eosin (HE), Periodic-acid Schiff (PAS) and Sirius red (SR) staining. Slides were processed for digitalization and quantification as previously described [18].

### 2.7. Microchimerism Evaluation

Theoretical microchimerism at infusion was estimated by dividing infused hepatocytes number by NMRI mouse hepatocellularity equivalent to (mean ± standard deviation (SD)) 109 ± 8 million hepatocytes/liver g individually adjusted for current liver weight extrapolated from relative liver weight at sacrifice. NMRI mouse hepatocellularity was calculated as described elsewhere [22], based on protein quantification of 9 NMRI mice livers and 2 NMRI mice hepatocyte suspensions. Mice LML and spleen were screened for microchimerism (male hepatocytes in female organ) based on predesigned droplet digital polymerase chain reaction (ddPCR) *Sry* (Mm.PT.58.28492124.g, Integrated DNA Technologies, Louvain, Belgium) and *Rpp30* (qMmuCEP0054985, Bio-Rad, Hercules, CA, USA) assays performed in multiplex on genomic DNA as described here [23]. *Sry* and *Rpp30* are located on chromosomes Y and 19, respectively. *Sry*/*Rpp30* copy number ratio is expected to be equal to 0.5 and 0 in male and female, respectively. Male microchimerism in female is equivalent to *Sry/Rpp30* copy number ratio multiplied by two because *Sry* is present in one copy (on Y chromosome) for each two *Rpp30* copies (on chromosomes 19).

### 2.8. General Statistical Analysis and Artwork

Kruskal-Wallis test with Dunns post-test with 95% confidence intervals were performed using GraphPad Prism v. 5.02 for Windows (GraphPad Software, San Diego, CA, USA). *p*-values < 0.05 were considered significant. When considered significant, Dunns post-test was reported on graph. Publisher (Office 365, Microsoft, Redmond, WA, USA) was used to draw figures.

## 3. Results

### 3.1. Hepatocyte Transplantation Was Safe and Well Tolerated by ZSD Mice

**Figure 2:**
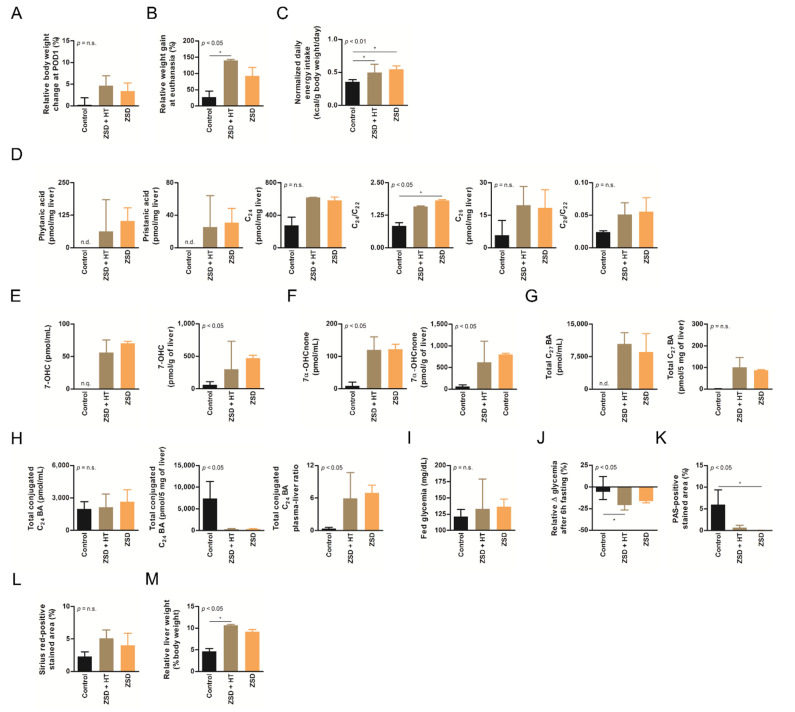
Low dose hepatocyte transplantation (HT) did not impact ZSD mice disease markers. (**A**) Relative body weight change at post-operative day (POD) 1, (**B**) relative weight gain at final follow-up, (**C**) normalized daily energy intake, (**D**) liver branched-chain and very long-chain fatty acids levels and ratio on C_22_, (**E**) plasma and liver oxysterols 7-hydroxycholesterol (7-OHC) and (**F**) 7α-hydroxycholestenone (7α-OHCnone) levels, (**G**) plasma and liver total C_27_ bile acids (BA) precursors levels, (**H**) plasma, liver and plasma-liver ratio of total conjugated C_24_ BA levels, (**I**) fed glycemia, (**J**) relative fasting glycemia drop (both fasting tests gave similar results and were merged on the graph, an outlier control mice was excluded from both tests), (**K**) relative liver Periodic-acid Schiff (PAS)-positive stained area (proxy for glycogen content), (**L**) relative liver Sirius red-positive stained area (proxy for collagen content) and (**M**) relative liver weight at sacrifice in control (*n* = 6), Zellweger spectrum disorder (ZSD) mice infused with low dose HT (*n* = 3) and ZSD mice sham operated (*n* = 2). HT outcome measures highlighted disease phenotype in ZSD mice but failed to detect low dose HT impact in treated mice. Kruskal-Wallis test, *n* (Control – ZSD + HT – ZSD) = 6 – 3 – 2 (except for **D**: *n* = 3 – 3 – 2 and **J**: *n* = 10 – 6 – 4), median ± interquartile range, *: *p*-value < 0.05, n.s.: not significant.

**Figure 3:**
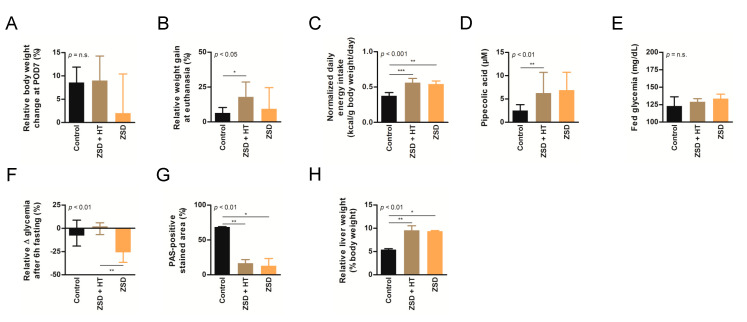
High dose hepatocyte transplantation (HT) was safe but failed to convincedly modify peroxisomal markers in ZSD mice. Relative body weight change at (**A**) post-operative day (POD) 7 or (**B**) sacrifice, (**C**) normalized daily energy intake, (**D**) plasma pipecolic acid levels, (**E**) fed glycemia, (**F**) relative fasting glycemia drop, (**G**) relative liver Periodic-acid Schiff (PAS)-positive stained area (proxy for glycogen content) and (**H**) relative liver weight in control (*n* = 6), Zellweger spectrum disorder (ZSD) mice treated with high dose HT (*n* = 8) and ZSD mice sham operated (*n* = 6). High dose HT protocol confirmed ZSD phenotype in affected mice and corrected fasting glycemia drop in ZSD mice but this was not correlated to increased liver PAS-positive stained area. Kruskal-Wallis test, *n* (Control – ZSD + HT – ZSD) = 6 – 8 – 6, median ± interquartile range, *: *p*-value < 0.05, **: *p*-value < 0.01, ***: *p*-value < 0.0001, n.s.: not significant.

In the next hours following HT, mice cleaned the surgical wound as detected by decreased povidone-iodine staining on post-operative day (POD) 1. To avoid suture traction, food kibbles were disposed on the litter during 24 h and mice started to eat them during POD1. Macroscopic liver evaluation at POD1 showed large necrosis area (Figure 1D) without clinical impact. During the post-HT period, none of the mice lost more than 5% body weight and none of the mice died (Figure 2A and Figure 3A). Food intake was not modified by HT (see below).

### 3.2. Growth and Normalized Daily Energy Intake Were Not Affected by HT in ZSD Mice

ZSD mice exhibit growth retardation despite higher normalized daily energy intake [18]. These anomalies were confirmed in this study and were not modified by low nor high dose HT (Figure 2B,C and Figure 3B,C).

### 3.3. HT Did Not Modify Plasma nor Liver Peroxisomal Marker Anomalies in ZSD Mice

ZSD mice show liver and plasma peroxisomal marker alterations classically found in ZSD patients [18]. In this study, liver BCFA were not detected in control mice and they did not seem affected by low dose HT in ZSD mice (Figure 2D). Liver VLCFA levels quantification did not highlighted significant accumulation in ZSD mice because of low sample size (Figure 2D). Plasma and liver oxysterols and BA levels were in accordance with our previous report and were not modified by low dose HT (Figure 2E–H). ZSD mice cholestasis was confirmed by total conjugated C_24_ BA plasma-liver ratio elevation, as previously reported [18], and it was not influenced by HT (Figure 2H).

Based on our experience of dramatic plasma pipecolic acid levels decrease shortly after LT and HT in ZSD patients [7,8], we chose to focus on this biochemical marker for the high dose HT protocol. As previously reported in our ZSD mouse model [18], plasma pipecolic acid levels were increased in ZSD mice. Unfortunately, high dose HT did not modify this parameter (Figure 3D).

### 3.4. Fasting Glycemia in ZSD Mice Was Improved after High Dose HT but this Was Not Correlated to Liver Glycogen Content

ZSD mice present glycemic alterations related to liver glycogen metabolism anomalies [18]. Fed glycemia was not significantly different between control and ZSD mice (Figure 2I and Figure 3E). Fasting glycemia was significantly lower in ZSD mice and this was rescued by high dose HT (Figure 2J and Figure 3F). Unfortunately, this was not correlated to liver PAS-positive stained area (a proxy for liver glycogen content [18]) which remained strongly lower in ZSD mice liver compared to control (Figure 2K and Figure 3G).

### 3.5. HT Did Not Reverse Relative Hepatomegaly in ZSD Mice

ZSD mice liver present a slight elevation in collagen deposition as measured by SR staining. This was not statistically evident in low dose HT ZSD mice partly because of the small sample size (Figure 2L). Another striking ZSD mice feature is their relative hepatomegaly [18]. This was confirmed in both HT protocols, but they failed to modify this hallmark of the disease (Figure 2M and Figure 3H).

### 3.6. Post-HT, Liver Histological Analysis Highlighted Spontaneously Resolving Necrosis and Confirmed ZSD Liver Pathology Hallmarks Compared to Controls

**Figure 4:**
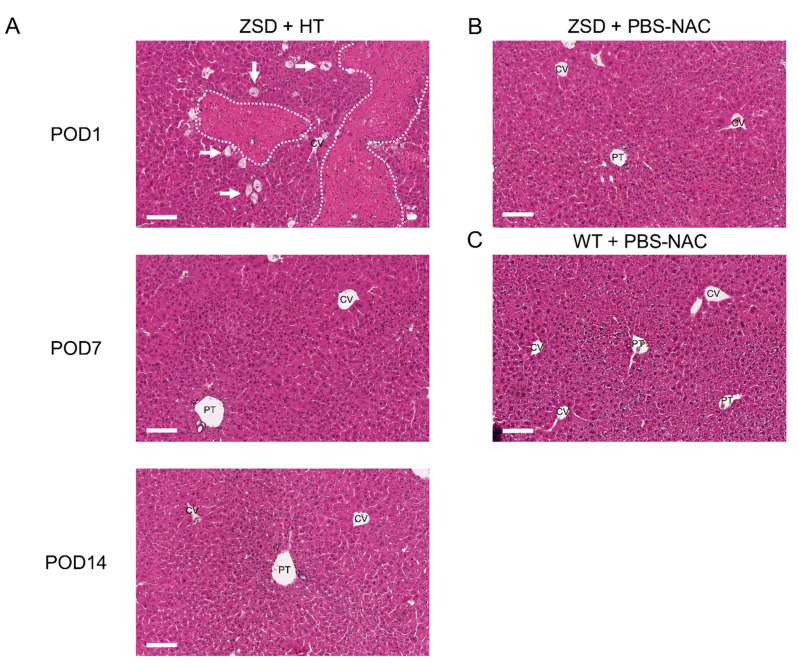
Liver histological analysis in Zellweger spectrum disorder (ZSD) mice confirmed ZSD liver disease hallmarks and uncovered spontaneously resolving focal liver necrosis following hepatocyte transplantation (HT). (**A**) Post-operative day (POD) 1 mice liver exhibited necrosis areas with inflammatory cells infiltrate (dotted lines) sparing the central vein (CV), along with ballooning degeneration of hepatocytes (arrows). POD7 and POD14 and (**B**) control (PBS-NAC) ZSD livers showed canalicular proliferation in portal tracts (PT) and progressive hepatocyte hypertrophy from PT to CV, as previously reported [18]. Necrosis was not detected at POD7 and POD14, nor in ZSD control livers, nor in (**C**) wild-type (WT) control livers. PBS-NAC: *N*-acetylcysteine in phosphate buffered saline. Scale bar: 100 μm.

To decipher post-HT engraftment, liver pathology analysis was performed on liver sections. Since the infused hepatocytes were isolated from a control littermate (i.e., a syngeneic donor), no specific histological marker could be used to localize the transplanted cells and engraftment was further measured by ddPCR (vide infra). As expected from previous report [24] and from the POD1 macroscopic liver evaluation (Figure 1D), POD1 liver HE staining in ZSD + HT mice showed necrosis areas with inflammatory cells infiltrate, sparing the central vein (CV) (Figure 4A). Liver necrosis resolved before POD7 and liver tissue architecture was preserved post-HT. ZSD liver displayed classical canalicular proliferation and progressive hepatocyte hypertrophy from portal tract (PT) to CV compared to wild-type control mice [18] (Figure 4B,C).

### 3.7. Male Liver Microchimerism Was Detected in Female ZSD Mice 24 h Post-HT and Was Lost during Follow-up

Theoretical liver microchimerism at infusion was (mean ± SD) 0.110 ± 0.003% (*n* = 3) and 0.461 ± 0.038% (*n* = 10) for low dose and high dose HT, respectively. *Sry*/*Rpp30* copy number ratio was measured by ddPCR on more than 35.000 droplets for each sample (Table 2). No liver microchimerism could be detected on low dose HT protocol samples (*n* = 2 females). At high dose HT POD1, liver microchimerism was 0.15%, which is equivalent to one third of the theoretical liver microchimerism at infusion (*n* = 1 female). After POD1, no significant liver microchimerism was detected (*n* = 5 females). For both protocols, no significant spleen microchimerism was detected by ddPCR (data not shown).

## 4. Discussion

To date, no validated treatment has been shown to modify the natural history of ZSD. Here, we report the evaluation of low dose versus high dose HT as therapeutic approach in a mild ZSD mouse model. We confirmed robustness and ZSD phenotype in *Pex1*-G844D homozygous mice. Both HT doses were safe despite the growth retardation affecting the ZSD mice. However, no long term hepatocyte engraftment could be detected. In consequence, no clinically relevant therapeutic effect was convincingly evidenced.

The first HT for liver-based metabolic disease were performed in animal models and patients devoid of liver injury phenotype [25,26,27]. Since then, successful HT protocols were reported in patients affected by chronic liver disease [28]. ZSD mouse models and patients exhibit chronic liver disease [18,29,30], and our team reported the safety of HT in a 4-year-old ZSD patient [8]. Our ZSD mouse model express a severe disease phenotype with growth retardation along with chronic liver injury and fibrosis [18,29]. Yet, this phenotype did not impede the feasibility of HT. This supports the safety of HT in ZSD, a disease associated with chronic liver disease.

Two third of the infused hepatocytes were lost 24 h post-HT, as published in rat and mouse models of HT [31,32]. In ZSD mice, toxic peroxisomal metabolites could potentially alter hepatocytes viability in the circulation before they reach liver sinusoids [33]. In addition, granulocytes, mononuclear cells [34] and Kupfer cells [35] seem to be a major player in exogenous cell clearance. Altering Kupfer cell phagocytic properties improves engraftment in HT rat model [35]. Moreover, HT triggers the instant blood-mediated inflammatory reaction (IBMIR), a process implicating the coagulation cascade which might explain the loss of transplanted hepatocytes [36]. In our model, NAC was used to prevent the procoagulant activity of the hepatocytes [20]. NAC might be insufficient and optimized anti-coagulation protocols should be evaluated for HT as it has been developed for liver-derived mesenchymal cells cell-based therapies [24,37] Finally, the size of mouse hepatocytes and sinusoids could account for the lower cell engraftment observed compared HT in rat models [38,39]. In a previous study, syngeneic male HT in female mice lead to 0.13–0.25% microchimersim 3–7 days after HT, but no longitudinal data are reported and HT protocol is lacking (i.e., mouse background, drug used to improve engraftment, etc.) [31].

A 4-year-old mild ZSD patient received 2 billion hepatocytes (10^8^ cells/kg of body weight, 2.5% theoretical microchimerism [27]) during a total of 5 days [8]. Male in female microchimerism measured on liver biopsy performed on the day of the last infusion reported 0.1–0.25% male cells [31]. In this child, hepatocyte loss during HT was equal to 90–95% as measured on last day of infusion. No long term hepatocyte engraftment was measured but biochemical parameters improved suggesting cell persistence [8]. Yet, after short term effect resulting probably from circulating cells (up to day 5) spontaneous trend to biochemical normalization cannot be excluded as it has been reported in some mild ZSD patients [40]. Compared to LT, HT biochemical impact is reduced in ZSD patients [7].

Poor hepatocyte engraftment in ZSD mice could be explained by the absence of selective advantage of the infused wild-type hepatocytes over ZSD hepatocytes. Hepatocyte proliferation was reported in ZSD mice liver but no necrosis was detected [18]. Absence of space for hepatocyte engraftment could be a major factor impairing HT in ZSD mouse liver. Moreover engraftment could be partly impaired in ZSD mice liver due to *Cxcl12* and *Hgf* downregulation (GSE145524), chemokines implicated in mesenchymal stromal cells recruitment and hepatocyte proliferation, respectively [41]. Several strategies have been developed to improve hepatocyte engraftment such as hepatotoxic drugs [35], preparative hepatic irradiation [42] combined or not with partial hepatectomy [25] and (repeated) reversible portal vein embolization [43]. The vast majority were evaluated in rat models of HT. In mice, uPA^+/+^-SCID mouse model combines liver insult leading to regenerative stimulus and immunosuppression for xenogenic liver cell therapy. Combining uPA^+/+^-SCID background to *Pex1*-G844D NMRI mouse was not attempted because it would potentially modify the robustness of the model [44]. Globally, only a fraction of these approaches are translatable into clinical practice and most of them are not devoid of risks [45].

Our study strengthened the ZSD phenotype of *Pex1*-G844D NMRI mouse model and demonstrated its suitability for therapeutic interventions like liver cell therapy in young light ZSD mice. Small sample size in low dose HT protocol limited the statistical significance of the results. Nevertheless, high dose HT protocol allowed us to conclude that hepatocyte engraftment was a barrier to therapeutic efficacy. In the future, techniques to improve liver cell engraftment (see above) or liver *Pex1* gene therapy could be evaluated in this mild ZSD mouse model to restore liver peroxisome biogenesis. Proof of concept was already evaluated by HT and LT in mild ZSD patients [7,8].

## 5. Conclusions

HT in *Pex1*-G844D NMRI mice was safe and confirmed the robustness and the ZSD phenotype. Both low dose and high dose HT failed to achieve long term hepatocyte engraftment. In consequence, no impact on disease markers was evidenced.

## Figures and Tables

**Table 1:** Hepatocyte Transplantation Protocol Description.

HT Protocol	Dose	HT (Age)	Sacrifice (Age)	Control Mice + PBS-NAC	ZSD Mice+ HT	ZSD Mice + PBS-NAC
Low dose HT	12.5 million/kg	4 weeks	8 weeks	6	3	2
High dose HT	50 million/kg	6 weeks	8 weeks	6	10 *	6

*: Mice were sacrificed after 1 day (*n* = 1) and 7 days (*n* = 1) for longitudinal microchimerism evaluation, and were excluded from HT outcomes analysis. HT: hepatocyte transplantation, PBS: phosphate buffered saline, NAC: *N*-acetylcysteine.

**Table 2:** Microchimerism evaluation on mice liver in low dose and high dose HT protocols.

Sample	Total Droplet Count	Mean *Sry* Concentration (Copy Number/µL)	Mean *Rpp30* Concentration (Copy Number/µL)	Mean *Sry*/*Rpp30* Ratio	Calculated Microchimerism
Male hepatocytes	52,789	464.750	896.750	0.518467	n.a.
No gDNA control	55,916	0	0	n.a.	n.a.
Low dose HT					
Male + PBS-NAC	36,195	507.333	1020.000	0.499494	n.a.
Female + PBS-NAC	48,151	0	945.667	0	n.a.
Female + HT	48,029	0	956.667	0	0%
Female + HT	38,277	0	1305.667	0	0%
High dose HT					
Male + PBS-NAC	51,464	513.500	1004.750	0.511389	n.a.
Female + PBS-NAC	52,453	0	1100.000	0	n.a.
Female + HT (POD1)	55,802	0.700	944.250	0.000748	0.150%
Female + HT (POD7)	54,368	0	913.500	0	0%
Female + HT	57,503	0	1002.250	0	0%
Female + HT	59,883	0.020 *	1043.250	0.000018	0.004%
Female + HT	55,344	0	939.500	0	0%
Female + HT	58,628	0	991.500	0	0%

Microchimerism was evaluated by droplet digital polymerase chain reaction (ddPCR) on genomic DNA (gDNA) extracted from female mouse liver and adequate controls (male hepatocytes, male liver and female liver sham infused). ddPCR was used to detect gDNA *Sry* (Y chromosome) and *Rpp30* (chromosome 19) copies in female mice infused with male mouse hepatocytes. *: based on one positive droplet. HT: hepatocyte transplantation, n.a.: not applicable, PBS-NAC: phosphate buffered saline and *N*-acetylcysteine (=sham), POD: post-operative day.

## Data Availability

The data presented in this study are available on request from the corresponding author.
